# Supplementary material for: Application of mechanical quantitative techniques in postoperative rehabilitation assessment of anterior cruciate ligament reconstruction: A study protocol
Source: PLoS One. 2025 Aug 6;20(8):e0324663. doi: 10.1371/journal.pone.0324663 (PMC12327682; doi:10.1371/journal.pone.0324663)
Supplement: S1 Appendix — (DOC) [file pone.0324663.s001.doc]

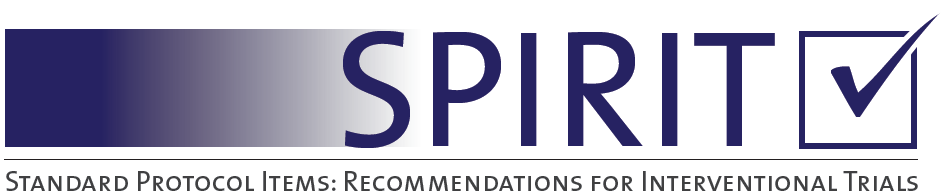


SPIRIT 2013 Checklist: Recommended items to address in a clinical trial protocol and related documents*

| Section/item | ItemNo | Description |
| --- | --- | --- |
| **Administrative information** | | |
| Title | 1 | Application of Mechanical Quantitative Techniques in Postoperative Rehabilitation Assessment of Anterior Cruciate Ligament Reconstruction:a study protocol |
| Trial registration | 2a | www.chictr.org.cn(PID:219889),Waiting for Review |
|  | 2b | All items of the World Health Organization Trial Registration Data Set are available in this record |
| Protocol version | 3 | 2025/3/17, 2.0 |
| Funding | 4 | Research Project of Hunan Provincial Sports Bureau  (2024KT0188) |
| Roles and responsibilities | 5a | n/a |
|  | 5b | n/a |
|  | 5c | This experiment is supported by the Hunan Provincial Sports Bureau (project number: [2024KT0188]), and the Hunan Rehabilitation Hospital is responsible for the supply of experimental materials and equipment. The specific role assignments are as follows:  1. Research Design  Funding participation level: not involved  Final decision-making power belongs to: Independent Academic Committee  2. Data management  Data collection: Researcher team  Database access permission: Open only to research teams  Data analysis: completed by independent statisticians  3. Publication of Achievements  Report writing: Written by the main researcher  Publication decision-making power: determined independently by the researcher  Achievement Attribution Agreement: Author Seats Allocated by Contribution |
|  | 5d | Test governance structure  1. Coordination Center  Composition: rehabilitation diagnosis and treatment center of Hunan rehabilitation hospital (including 1 project director, 2 research coordinators and 1 quality control specialist)  Responsibilities:  ▪ Version control of test documents  ▪ Inter center standardized training  ▪ 24-hour response process for serious adverse events (SAE)  2. Steering Committee  ▪ Composition: 7 independent members (3 Methodists, 2 clinical experts, 1 statistician, 1 ethicist)  ▪ Decision making mechanism: quarterly meeting (with schedule of 2023 annual meeting)  ▪ Authority: have the right to terminate the participation of the sub center that does not conform to the declaration of Helsinki  3. endpoint determination Committee  Declaration of Independence: all members have no economic interest with the sponsor  Workflow:  ▪ Three level ruling system (primary interpretation → dispute marking → plenary voting)  ▪ Use standardized CRF decision table  4. data management team  System architecture: redcap electronic data acquisition system  Quality control:  ▪ Double entry verification (error rate<0.5%)  ▪ Generate data integrity report weekly  ▪ Independent data monitoring committee (DMC) verification authority  5. other regulatory bodies  DSMB: composed of 3 clinical pharmacologists who are not research participants  Ethics and compliance office: responsible for the standardization of signing the informed consent form of audit every six months |
| Introduction |  |  |
| Background and rationale | 6a | 1.1Anterior Cruciate Ligament Injury is Becoming an Increasingly Significant Health Issue  Anterior cruciate ligament (ACL) injuries are relatively common sports injuries among athletes, posing a severe threat to their competitive performance and athletic careers [1]. In the United States, for instance, over 350,000 athletes and sports enthusiasts annually experience ACL injuries [2], with approximately half necessitating surgical intervention [3]. The ACL's physiological attributes impart it with limited self-repair capabilities, leading to the clinical consensus that complete (grade III) ACL tears necessitate anterior cruciate ligament reconstruction (ACLR) [4]. The primary objective of ACLR is to reinstate knee joint stability and postpone the progression of osteoarthritis [5]. Despite significant advancements in surgical techniques for ACL injuries, existing literature indicates a substantial risk of second ACL ruptures post-ACLR, with a prevalence rate as high as 25% among athletes [6]. This elevated risk is primarily attributed to inadequate functional recovery and biomechanical stability post-surgery, rendering the knees vulnerable during high-impact activities [7]. Therefore, more scientific and precise assessment methods are needed post-ACLR to ensure athletes can safely return to sports and reduce the risk of a second ACL rupture.  1.2The Importance of Knee Joint Function Assessment  Knee function assessment plays a pivotal role in the management and rehabilitation of patients with anterior cruciate ligament (ACL) injuries. Accurate evaluation enables clinicians to determine the severity of ACL injuries, providing a critical foundation for the development of personalized treatment strategies. During rehabilitation, routine assessments not only facilitate the monitoring of patient progress and ensure the efficacy of the rehabilitation protocols but also enable the early detection and intervention of potential complications such as muscle atrophy and joint stiffness. Furthermore, periodic evaluation improve patient awareness of their recovery status, fostering greater confidence in treatment, enhancing adherence to rehabilitation programs, and ultimately improving overall quality of life.  Currently, knee function assessment primarily relies on clinical rating scale. While these scales are widely adopted due to their simplicity and ease of administration, their reliability is often compromised by inter-individual variability, assessor subjectivity, and dependence on clinical experience. This limitation is particularly evident in muscle tone assessment, where obtaining objective quantitative data remains challenging [8]. Several studies suggest that conventional spasticity assessment scales fail to accurately reflect joint angular velocity or range of motion [10]. Similarly, manual muscle testing is inherently subjective. Although isokinetic strength testing is considered the "gold standard" for muscle strength quantification, its clinical application is constrained by the requirement for costly, bulky |
|  |  | equipment and the logistical challenges associated with patient positioning and transfer [9]. Imaging modalities constitute another essential component of knee function assessment. Traditional imaging techniques such as X-ray and computed tomography (CT) provide high-resolution visualization of bone morphology; however, their two-dimensional nature limits the accurate representation of soft tissue structures [12]. Additionally, these modalities present inherent risks of radiation exposure and may cause pain during the examination process [11]. While magnetic resonance imaging (MRI) offers superior diagnostic accuracy for soft tissue evaluation, its clinical utility is limited by the necessity for multi-sequence, multi-plane scanning, which significantly increases healthcare costs and requires advanced technical expertise. Moreover, MRI is unsuitable for real-time dynamic assessment of functional movement. Given these limitations, there is a compelling clinical need for the development of more precise, real-time, and objective methodologies for comprehensive knee function assessment.  1.3Application of Musculoskeletal Ultrasound in the Assessment of Anterior Cruciate Ligament Injuries  In recent years, musculoskeletal ultrasound has emerged as a focal point in clinical research for knee joint function diagnosis. It is non-invasive, real-time, convenient, and radiation-free [11], offering advantages such as ease of operation and low cost, which have contributed to its widespread clinical application [13]. Musculoskeletal ultrasound provides a clear visualization of the structural characteristics of the knee joint, allowing dynamic observation of muscles and tendons [14]. It enables precise assessment of anatomical abnormalities and functional changes in the knee joint, serving as a valuable reference for personalized rehabilitation treatment [15].  Shear wave elastography (SWE), a recently developed technology, enhances traditional ultrasound capabilities by sensitively detecting changes in tendon hardness. Building upon high-frequency ultrasound, SWE more effectively identifies minor tears [16] and objectively evaluates muscle tissue hardness variations [17]. However, despite its advantages, SWE technology faces several limitations in clinical application. Operating SWE requires experience with two-dimensional ultrasound [18], hindering its broader adoption. Additionally, measurement results can vary across imaging devices from different manufacturers, with studies showing stastistically significant differences in shear wave speed (SWS) [19]. Furthermore, the calculation model for SWE assumes the test subject is a uniform, isotropic elastic body [20], yet musculoskeletal tissue exhibits clear anisotropic properties, failing to meet the conditions of Young's modulus formula. The order of magnitude changes in Young's modulus after conversion further increase measurement variability [21]. Some researchers even suggest that SWE data presents extraction and interpretation challenges, with unclear physical significance [22].  1.4Advantages and Research Progress of Mechanical Quantitative Techniques in the Assessment of Anterior Cruciate Ligament Injuries  The propagation characteristics of waves in biological soft tissues under dynamic mechanical loads demonstrate intricate behaviors that are closely linked to the tissues' mechanical properties. In particular, the velocity of shear wave propagation serves as a key indicator of tissue stiffness: the faster the wave propagates, the stiffer the tissue.[23]. By meticulously analyzing the in vivo propagation patterns of different wave modes, researchers can quantitatively characterize the mechanical properties of soft tissues. This approach enables a non-invasive and non-destructive assessment of both physiological and pathological states of human soft tissues, providing new perspectives for disease diagnosis, prognosis, and broader public health implications. Due to the inherent variability in human tissues properties, precisely characterizing their mechanical behavior remains a major challenge in biomechanics. |
|  |  | Building upon these wave propagation principles, quantitative musculoskeletal biomechanics assessment integrates sophisticated biomechanical modeling with the critical clinical challenge of knee joint function evaluation in patients with ACL injuries. By employing non-destructive quantitative biomechanical measurements, this approach establishes direct quantitative correlation between the degree of knee joint functional recovery and the mechanical state of skeletal muscles in ACL-injuried patients. Consequently, it introduces a novel clinical evaluation framework for knee joint recovery that overcomes the subjectivity of traditional grading scales, thereby providing a more precise and objective assessment. Moreover, the musculoskeletal biomechanical measurement device is user-friendly, non-invasive, and painless, making it suitable for rehabilitation assessments across all age groups. The data and findings from this research are expected to significantly advance rehabilitation protocols and establish evidence-based guidelines for the clinical management of ACL injury patients. |
|  | 6b | n/a |
| Objectives | 7 | This study protocol aims to explore the application of mechanical quantitative techniques in the postoperative rehabilitation assessment following anterior cruciate ligament reconstruction (ACLR). |
| Trial design | 8 | This study primarily employs an observational approach, focusing on musculoskeletal mechanical quantitative detection technology, and includes the following steps: Firstly, collect clinical data from patients with anterior cruciate ligament injuries, including medical history, imaging examinations, etc., to ensure the diversity and representativeness of the sample. Secondly, use musculoskeletal mechanical quantitative measurement devices to conduct quantitative, non-destructive measurements of the patient's knee joint to obtain relevant parameters of the mechanical state of skeletal muscles. During the measurement process, strictly adhere to operational protocols to ensure the accuracy and reliability of the data. Then, conduct traditional rehabilitation assessments of the knee joint. Concurrently, combine quantitative modeling and characterization methods of biomechanics to deeply analyze the measurement data, revealing the quantitative correlation between the degree of knee joint functional recovery in patients with anterior cruciate ligament injuries and the results of traditional rehabilitation assessments. Finally, based on the analysis results, propose a new clinical evaluation method for knee joint functional recovery and conduct validation and assessment. Additionally, collect subjective feedback from patients to further verify the accuracy and feasibility of the new evaluation method. |
| Methods: Participants, interventions, and outcomes | | |
| Study setting | 9 | This study will be conducted in Hunan Provincial rehabilitation hospital and several hospitals and community clinics in Hunan Province to ensure the wide applicability and representativeness of the  research results. The study will only be conducted in China, and the participating institutions have the ability and representativeness in the field of rehabilitation assessment and treatment. The complete list of research sites will be provided through the official website of the Research Coordination Center for researchers and participants to query |
| Eligibility criteria | 10 | a.Inclusion criteria:  ①Unilateral leg ACL injury, arthroscopic autologous hamstring or patellar tendon ACL  reconstruction surgery, with no other ligamentous injuries involved;  ②Swelling of the affected knee joint is grade 0 or 1+;  ③Time from injury to surgery is less than 2 months;  ④Ages between 18 and 60 years;  ⑤Signed an informed consent form for rehabilitation treatment and actively cooperate with the  treatment;  ⑥The treatment plan is approved by the Ethics Committee of the Capital University of Physical  Education and Sports;  b.Exclusion criteria  ①Concurrent posterior cruciate ligament rupture or meniscal repair;  ②Concurrent postoperative complications affecting limb exercise;  ③Patients with a history of hip joint trauma;  ④Individuals with hypertension, diabetes, or other chronic diseases of organs;  ⑤Individuals with concurrent severe diseases of the heart, brain, kidneys, and hematopoietic  system, and patients with mental illnesses;  ⑥Concurrent with any conditions that are detrimental to patient recovery or continuation of the  trial. |
| Interventions | 11a | Utilize a musculoskeletal mechanical quantitative detector (M5) to measure the mechanical quantitative characteristics (shear modulus G) of the subject's rectus femoris and hamstring muscles. |
|  | 11b | Criteria for discontinuing or modifying allocated interventions for a given trial participant (eg, drug dose change in response to harms, participant request, or improving/worsening disease) |
|  | 11c | Strategies to improve adherence to intervention protocols, and any procedures for monitoring adherence (eg, drug tablet return, laboratory tests) |
|  | 11d | Relevant concomitant care and interventions that are permitted or prohibited during the trial |
| Outcomes | 12 | Primary：Musculoskeletal Mechanical Quantitative Assessment: A physician with over 5 years of  experience is designated to conduct musculoskeletal mechanical quantitative assessments, The  assessment includes musculoskeletal mechanical quantitative testing:①Shear modulus of the  rectus femoris muscle (modulus of rigidity, G);②Shear modulus of the hamstring muscles  (modulus of rigidity, G).  Secondary1:Knee Joint Muscle Strength Grading: During the examination, the patient is placed in  different positions to be tested, and the targeted muscles or muscle groups perform specific  movements under conditions of weight reduction, gravity resistance, or resistance, achieving the  maximum range of motion. Based on the muscle's ability to perform the movement, muscle  strength is graded according to the grading standards, which are mainly divided into 6 levels: 0, 1, 2, 3, 4, and 5. Level 5 represents normal strength.  Secondary2:Lysholm Score:①Pain Score: 0-25 points.②Instability Score: 0-25 points.③Squatting Score:  0-5 points.④Locking Score: 0-15 points.⑤Climbing Stairs Score: 0-10 points.⑥Support Score:  0-5 points.⑦Swelling Score: 0-10 points.⑧Gait Score: 0-5 points. |
| Participant timeline | 13 | Time schedule of enrolment, interventions (including any run-ins and washouts), assessments, and visits for participants. A schematic diagram is highly recommended (see Figure) |
| Sample size | 14 | In this study, the Lysholm knee score questionnaire comprises a total of 8 variables, basic functional assessment includes 2 variables: muscle strength and range of motion, mechanical quantitative assessment results include 2 variables, a total of 12 research variables are included in this study, referring to Kendall's sample size estimation method, the sample size included is 5 to 10 times the number of variables, considering a 10% rate of ineligible cases, the total sample size should be at least 66 cases, according to the actual situation, the final sample size is determined to be 66 knee joints. |
| Recruitment | 15 | The research subjects will be recruited through the official website of Hunan Provincial rehabilitation hospital, and the recruitment announcement will be published in Hunan Provincial rehabilitation hospital. Researchers will receive training in communication with potential subjects and their relatives, document preparation including screening logs, and other standard operating procedures. The expected recruitment time is 4-6 weeks. |
| **Methods: Assignment of interventions (for controlled trials)** | | |
| Allocation: |  |  |
| Sequence generation | 16a | n/a.In a case-control design, the traditional rehabilitation assessments implemented for the knee  joint include: 1. Knee joint muscle strength; 2. Range of motion (ROM) of the knee joint; 3. Lysholm score. The mechanical quantitative assessment team conducts mechanical quantitative  evaluations on the knee joint. |
| Allocation concealment mechanism | 16b | n/a.In a case-control design, there will not be concealed information to the participant. |
| Implementation | 16c | The research team is composed of doctors, physiotherapists, data managers and Statistics experts. The team members have been strictly screened and have rich clinical experience and research  ability. Doctors were responsible for the recruitment of participants, medical history collection and quantitative mechanical evaluation. Physiotherapists are responsible for traditional knee function  assessment (including shoulder range of motion measurement, strength assessment, Lysholm scale score, etc.) and follow-up work. Data management personnel are responsible for data collection, entry, sorting and data analysis. All data will be managed through the electronic data capture system (EDC) to ensure data integrity and security. |
| Blinding (masking) | 17a | n/a.Due to the particularity of clinical controlled trials, strict double-blind trials cannot be carried out. Therefore, according to the characteristics of this study, the participants knew the evaluation content, but the evaluators did not know the specific research objectives and other team evaluation results. Set data entry or statistics grouping personnel independently. |
|  | 17b | n/a.There will be only assessors being blinded in this research, so unblinding will not occur. |
| **Methods: Data collection, management, and analysis** | | |
| Data collection methods | 18a | Clinical investigators and trial participants input the data into the electronic case report form (ECRF). The muscle modulus value g will be evaluated and recorded by special personnel through mechanical quantitative technology and equipment. An independent group was set up to evaluate the knee strength, range of motion and Lysholm scale. |
|  | 18b | Doctors, physiotherapists and other researchers will carefully take care of the test participants until the last follow-up. During the test, researchers will answer any test participants' questions about the  study. In addition, patients will also be informed that there is scientific evidence that the evaluation results received in this clinical trial may be more accurate than those received under conventional circumstances. |
| Data management | 19 | This study plans to use electronic case report form (ECRF) to complete data collection and storage. Researchers will be trained in system operation and data entry before recruiting the first patient. Only authorized research members can access the research database. Based on the functional roles of the research members in the research, the corresponding forms will be customized for authority allocation. |
| Statistical methods | 20a | The central accomplishment of this research has been to establish and confirm the correlation between the outcomes of mechanical quantitative technology assessments and traditional functional  evaluation methods. These traditional methods include assessments of knee muscle strength, range of motion (ROM), and Lysholm scores. To analyze the primary outcomes, the researchers compared the evaluation values of knee joint function with the mechanical quantitative modulus values. They then conducted a detailed analysis  of the results from both sets of assessments. For these statistical analyses, the research team used SPSS version 20.0 software to create a comprehensive database. Categorical data were presented as frequencies and percentages, with group comparisons made  using chi-square tests or exact probability methods. Continuous data were expressed as means ±standard deviations, and group comparisons were conducted using t-tests, analysis of variance  (ANOVA), or rank sum tests. To explore the correlation between different factors, the researchers employed Spearman's correlation coefficient. A p-value of less than 0.05 was set as the threshold for statistical significance. Throughout the process, data entry and statistical grouping were managed by independent personnel to ensure objectivity and accuracy. This approach helped maintain the integrity of the data and the reliability of the findings |
|  | 20b | There are no subgroups in this protocol, but we will analyze the impact of different degrees of knee dysfunction on the primary and secondary outcomes of the body |
|  | 20c | Handling Protocol Non-Adherence  To handle potential deviations from the study (e.g., missed assessments, non-compliance with research protocols, or unplanned interventions), the following strategies will be implemented:  Intention-to-Treat (ITT) Analysis:All participants in the study will be included in the preliminary analysis regardless of adherence to the protocol. This method can minimize the bias introduced by  selective exclusion. Per-Protocol (PP) Sensitivity Analysis: A secondary analysis will be conducted on participants who strictly abide by the study protocol. This will assess the stability of the main results and assess whether deviations from the scheme significantly affect the results. Documentation and Reporting: Detailed records of protocol deviations will be maintained (e.g., missed meetings, incomplete assessments). The frequency, causes and potential impacts of non- compliance will be reported transparently in the final analysis. Handling Missing Data Missing data resulting from participant dropout, missed follow-ups, or incomplete measurements  will be managed through the following methods:  Multiple Imputation (MI): For continuous variables (e.g., mechanical modulus values, Lysholm scores), missing data will be imputed using MI with predictive mean matching. Five imputed datasets will be generated, and pooled estimates will be derived using Rubin’s rules. Sensitivity to the missing-at-random (MAR) assumption will be tested by comparing results across imputed and complete-case datasets. Complete-Case Sensitivity Analysis: A secondary analysis will exclude participants with missing data to evaluate whether conclusions align with the primary MI-based results. Multiple Imputation (MI)：For continuous variables, such as mechanical modulus values and  Lysholm scores, missing data will be handled using Multiple Imputation (MI) with predictive mean matching. Specifically, five imputed datasets will be generated. Pooled estimates will then be  derived according to Rubin’s rules. The sensitivity of our analyses to the missing-at-random (MAR) assumption will be assessed by comparing results across the imputed datasets and the complete-case  dataset. Complete-Case Sensitivity Analysis：A secondary analysis will be conducted, excluding participants with any missing data, to determine whether the conclusions drawn align with those  from the primary analysis using MI. This approach will help evaluate the robustness of our findings to potential biases introduced by missing data. |
| **Methods: Monitoring** | | |
| Data monitoring | 21a | Since the research will only evaluate the participants without treatment or intervention, it can be considered as low-risk, so the data monitoring committee is not considered. |
|  | 21b | n/a.No interim analyses will be needed. |
| Harms | 22 | Adverse events of any type were systematically collected throughout the research. In the event of an emergency, these events were immediately reported to the chief expert and documented in a  dedicated adverse event file. The chief expert is responsible for managing adverse events and, when necessary, referring participants to appropriate departments or clinicians for further evaluation and  treatment. Any serious adverse events (SAEs) will be promptly reported to the ethics committee. |
| Auditing | 23 | n/a.No regulatory authority audit is planned for this trial. The methods employed in this trial are  considered to be low-risk. |
| Ethics and dissemination | | |
| Research ethics approval | 24 | This study has been approved by the ethics committee of Hunan rehabilitation hospital(ID:2024101101) |
| Protocol amendments | 25 | If significant revisions to the research protocol are necessary, the updated content will be communicated to the ethics committee, the trial registry, and the journal to which the study is submitted. |
| Consent or assent | 26a | The investigator should explain the details of the clinical trial to the subjects or guardians adequately, including the known and foreseeable risks and possible adverse events. Considering  that some patients may lack understanding of their own diseases, the subjects or their guardians and researchers should sign and date the informed consent form after full and detailed explanation. This study has been declared and reviewed by the ethics committee. It is considered that participating in the clinical trial is in the subject's own interests. Patients can enter the clinical trial only after their guardian signs, and their guardian signs and indicates the date before being included in the trial. |
|  | 26b | n/a. Biological specimens will not be collected in this trial. |
| Confidentiality | 27 | This study strictly complies with the General Data Protection Regulation (GDPR), guaranteeing the confidentiality and security of participants' data. All collected data will be pseudonymized and will not be stored on any server. De-identified data will be encoded using the initials of participants' Chinese name phonetic pronunciation for analysis. The study adheres to the ethical guidelines of the World Medical Association's Declaration of Helsinki, with data access limited exclusively to the principal researchers. |
| Declaration of interests | 28 | The authors declare no competing interests. |
| Access to data | 29 | Access to the final dataset will be restricted to the lead investigator and the corresponding author. The corresponding author will provide the relevant data upon reasonable request. |
| Ancillary and post-trial care | 30 | n/a. There will not be anticipated harm ,discomfort and compensation to the participation. This study focuses on assessment and testing of knee function. There is no provision for post-trial care. |
| Dissemination policy | 31a | Upon completion of data collection, the statistician will prepare a comprehensive statistical report detailing the primary research findings. The research team will convene to discuss these results, after which they will draft a research manuscript. This manuscript will be submitted for peer review and subsequently published in leading scientific journals. Additionally, efforts will be made to present the findings at prominent conferences in the field of rehabilitation medicine |
|  | 31b | The protocol adheres to the International Committee of Medical Journal Editors (ICMJE) criteria for authorship eligibility. Authors must have made substantial contributions to the conception, design, acquisition, analysis, or interpretation of data; drafted the work or substantively revised it; approved the submitted version; and agreed to be accountable for all aspects of the work. All authors must meet these criteria, and no individual who does not meet them will be listed as an author.  Professional writers may be engaged to assist in the preparation of the manuscript, but only if they are appropriately acknowledged and their contributions are transparently disclosed. Any professional writer used will have relevant expertise in the field and will follow ethical guidelines for collaborative writing. The funding source for any professional writer will be clearly stated in the manuscript.  The protocol will ensure that all contributors, including professional writers, are appropriately recognized and that the integrity of the research and reporting process is maintained. |
|  | 31c | Plans, if any, for granting public access to the full protocol, participant-level dataset, and statistical code |
| Appendices |  |  |
| Informed consent materials | 32 | All researchers have provided their consent for publication. The informed consent materials of trial participants are available from the corresponding author upon reasonable request. |
| Biological specimens | 33 | n/a.No biological specimens will be collected in this research. |

*It is strongly recommended that this checklist be read in conjunction with the SPIRIT 2013 Explanation & Elaboration for important clarification on the items. Amendments to the protocol should be tracked and dated. The SPIRIT checklist is copyrighted by the SPIRIT Group under the Creative Commons “[Attribution-NonCommercial-NoDerivs 3.0 Unported](http://www.creativecommons.org/licenses/by-nc-nd/3.0/)” license.
