# Supplementary material for: Application of mechanical quantitative techniques in postoperative rehabilitation assessment of anterior cruciate ligament reconstruction: A study protocol
Source: PLoS One. 2025 Aug 6;20(8):e0324663. doi: 10.1371/journal.pone.0324663 (PMC12327682; doi:10.1371/journal.pone.0324663)
Supplement: S2 Appendix — (DOCX) [file pone.0324663.s002.docx]

Figure1. Application of Mechanical Quantitative Techniques in Postoperative Rehabilitation Assessment of Anterior Cruciate Ligament Reconstruction:a study protocol

|  | **STUDY PERIOD** | | | | | | | |
| --- | --- | --- | --- | --- | --- | --- | --- | --- |
|  | **Enrolment** | **Allocation** | **Post-allocation** | | | | | **Close-out** |
| **TIMEPOINT**** | ***-t_1_*** | **0** | ***t_1_*** | ***t_2_*** | ***t_3_*** | ***t_4_*** | ***etc.*** | ***t_x_*** |
| **ENROLMENT:** | X |  |  |  |  |  |  |  |
| **Eligibility screen** | X |  |  |  |  |  |  |  |
| **Informed consent** | X |  |  |  |  |  |  |  |
| ***Baseline*** |  | X |  |  |  |  |  |  |
| **Allocation** |  | X |  |  |  |  |  |  |
| **INTERVENTIONS:** |  |  |  |  |  |  |  |  |
| ***Interventions*** |  |  |  |  |  |  |  |  |
| **ASSESSMENTS:** |  |  |  |  |  |  |  |  |
| ***MMT*** |  |  | X |  |  |  |  | X |
| ***ROM*** |  |  | X |  |  |  |  | X |
| ***Lysholm score*** |  |  | X |  |  |  |  | X |
| ***G*** |  |  | X |  |  |  |  | X |

*Recommended content can be displayed using various schematic formats. See SPIRIT 2013 Explanation and Elaboration for examples from protocols.

**List specific timepoints in this row.
