# Supplementary material for: Application of mechanical quantitative techniques in postoperative rehabilitation assessment of anterior cruciate ligament reconstruction: A study protocol
Source: PLoS One. 2025 Aug 6;20(8):e0324663. doi: 10.1371/journal.pone.0324663 (PMC12327682; doi:10.1371/journal.pone.0324663)
Supplement: S3 Appendix — (PDF) [file pone.0324663.s003.pdf]

Application of Mechanical Quantitative Techniques in Postoperative Rehabilitation Assessment of Anterior Cruciate Ligament Reconstruction：a study protocol

本研究的完整细节和计划已经由湖南省康复医院医学伦理委员会审查通过，项目编号：2024101101
The the complete and detailed plan of this research has been approved by the Ethics Committee of Hunan Provincial Rehabilitation Hospital(Approval No.:2024101101)

1. Materials and methods

1.1 Clinical trials

This study primarily employs an observational approach, focusing on musculoskeletal mechanical quantitative detection technology, and includes the following steps: Firstly, collect clinical data from patients with anterior cruciate ligament injuries, including medical history, imaging examinations, etc., to ensure the diversity and representativeness of the sample. Secondly, use musculoskeletal mechanical quantitative measurement devices to conduct quantitative, non-destructive measurements of the patient's knee joint to obtain relevant parameters of the mechanical state of skeletal muscles. During the measurement process, strictly adhere to operational protocols to ensure the accuracy and reliability of the data. Then, conduct traditional rehabilitation assessments of the knee joint. Concurrently, combine quantitative modeling and characterization methods of biomechanics to deeply analyze the measurement data, revealing the quantitative correlation between the degree of knee joint functional recovery in patients with anterior cruciate ligament injuries and the results of traditional rehabilitation assessments. Finally, based on the analysis results, propose a new clinical evaluation method for knee joint functional recovery and conduct validation and assessment. Additionally, collect subjective feedback from patients to further verify the accuracy and feasibility of the new evaluation method.All trial procedures and radiological and clinical visits are summarized in Figure 1a, b.This research has been registered in OSF (DOI:doi.org/10.17605/OSF.IO/K24QV) . And the Ethics Committee of Hunan Provincial Rehabilitation Hospital has approved this research at 11 October 2024 (Approval No.:2024101101).

Figure 1a　　Figure 1b

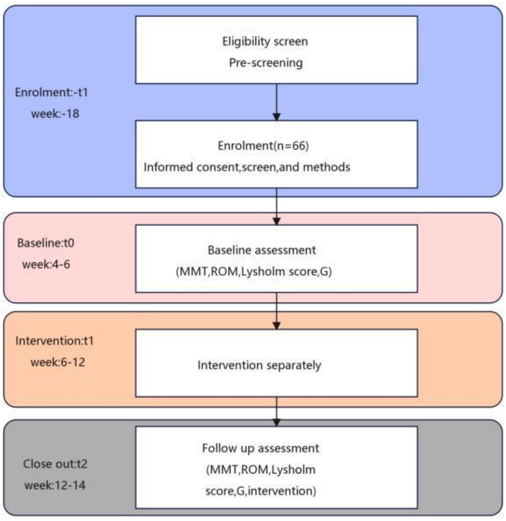

Figure1. Application of Mechanical Quantitative Techniques in Postoperative Rehabilitation Assessment of Anterior Cruciate Ligament Reconstruction:a study protocol

	STUDY PERIOD							
	Enrolment	Allocation	Post-allocation					Close-out
TIMEPOINT**	$-t_1$	0	t_1	t_2	t_3	t_4	etc.	t_x
ENROLMENT:	X							
Eligibility screen	X							
Informed consent	X							
Baseline		X						
Allocation		X						
INTERVENTIONS:								
Interventions				←	—	→		
ASSESSMENTS:								
MMT			X					X
ROM			X					X
Lysholm score			X					X
G			X					X

*Recommended content can be displayed using various schematic formats. See SPIRIT 2013 Explanation and Elaboration for examples from protocols.
**List specific timepoints in this row.

1.2 Experimental study design

1.2.1 Sample recruitment

Inclusion criteria:

①Unilateral leg ACL injury, arthroscopic autologous hamstring or patellar tendon ACL reconstruction surgery, with no other ligamentous injuries involved;

②Swelling of the affected knee joint is grade 0 or 1+;

③Time from injury to surgery is less than 2 months;

④Ages between 18 and 60 years;

⑤Signed an informed consent form for rehabilitation treatment and actively cooperate with the treatment;

⑥The treatment plan is approved by the Ethics Committee of the Hunan Provincial Rehabilitation Hospital;

Exclusion criteria

①Concurrent posterior cruciate ligament rupture or meniscal repair;

②Concurrent postoperative complications affecting limb exercise;

③Patients with a history of hip joint trauma;

④Individuals with hypertension, diabetes, or other chronic diseases of organs;

⑤Individuals with concurrent severe diseases of the heart, brain, kidneys, and hematopoietic system, and patients with mental illnesses;

⑥Concurrent with any conditions that are detrimental to patient recovery or continuation of the trial.

Sample size

In this study, the Lysholm knee score questionnaire comprises a total of 8 variables, basic functional assessment includes 2 variables: muscle strength and range of motion, mechanical quantitative assessment results include 2 variables, a total of 12 research variables are included in this study, referring to Kendall's sample size estimation method, the sample size included is 5 to 10 times the number of variables, considering a 10% rate of ineligible cases, the total sample size should be at least 66 cases,

according to the actual situation, the final sample size is determined to be 66 knee joints.

1.2.2 Implementation method

(1)Data Statistics and Analysis:①Firstly, a designated person evaluates the cases that meet the inclusion criteria and records the baseline data; ② One assessment is conducted during the rehabilitation process and after treatment, with proper data recording;③Finally, a professional separate from the assessment work performs data statistics and analysis.(2) Knee Joint Function Assessment: A fixed experienced intermediate rehabilitation therapist conducts the functional assessment, which includes: knee joint muscle strength, ROM, and Lysholm score.

(2) Knee Joint Muscle Strength Grading: During the examination, the patient is placed in different positions to be tested, and the targeted muscles or muscle groups perform specific movements under conditions of weight reduction, gravity resistance, or resistance, achieving the maximum range of motion. Based on the muscle's ability to perform the movement, muscle strength is graded according to the grading standards, which are mainly divided into 6 levels: 0, 1, 2, 3, 4, and 5. Level 5 represents normal strength.

Lysholm Score: ① Pain Score: 0-25 points. ② Instability Score: 0-25 points. ③ Squatting Score: 0-5 points.④Locking Score: 0-15 points.⑤Climbing Stairs Score: 0-10 points.⑥Support Score: 0-5 points.⑦Swelling Score: 0-10 points.⑧Gait Score: 0-5 points.

(3) Musculoskeletal Mechanical Quantitative Assessment: A physician with over 5 years of experience is designated to conduct musculoskeletal mechanical quantitative assessments, The assessment includes musculoskeletal mechanical quantitative testing:①Shear modulus of the rectus femoris muscle (modulus of rigidity, G);②Shear modulus of the hamstring muscles (modulus of rigidity, G).

Specific operation method:

a: Measurement Method: Utilize a musculoskeletal mechanical quantitative detector (M5) to measure the mechanical quantitative characteristics (shear modulus G) of the subject's rectus femoris and hamstring muscles.

b: Subject Position: The patient is in a supine position with both lower limbs relaxed.

c: Operating SOP(Standard Operating Procedure):

Protocol: Experimental Equipment, Consumables, and Paper Documents:

① Experimental Equipment: Musculoskeletal mechanical quantitative detector, including the main unit, 9L3-8.5MHZ linear array transducer, and mechanical excitation module.

②Consumables: Ultrasound coupling gel.

③Paper Documents: Informed consent form for subjects, subject information collection form, and experimental record form.

④Other Experimental Tools and Consumables (including a standard medical bed, measuring tape, power strip, ultrasound-specific paper, marker, heat patch, storage box, low stool, etc.).

Pre-experimental Preparation:

①Coupling Gel Preheating: One hour before the official start of the experiment,

attach a heat patch to the coupling gel bottle to warm it to a temperature similar to body temperature for the formal experiment;
②Paper Document Preparation: Print paper documents according to the number of subjects, one per person;
③Subject Informed Consent: The experimenter informs the subjects about the experimental process, benefits, risks, confidentiality plan, etc., and after being fully informed, the subjects sign the informed consent form;
④Subject Completes Information Collection Form;
Instrument Preparation
①Place the musculoskeletal mechanical quantitative detector and its accessories on the experimental table, ensuring the main unit is properly connected to the transducer; place the power strip in the experiment box, connect the main unit to the power strip. Press and hold the power button on the side of the musculoskeletal mechanical quantitative detector until the power indicator light turns on, open the app, and confirm login.
②Tap the E button on the left sidebar of the main unit screen to enter the force-sound acquisition mode, and check the parameters as shown in the table 1 below:

Table 1. Parameters

Parameter	Value
Frequency	6.0M
Line Count	4
Range	5mm
Time	300ms
Position Preset	2
Display Line Number	Off

③Adjust the depth position of the measurement point caliper to set the starting point of the acquisition depth range just below the dermis layer under the transducer as shown in the figure 2:

Figure 2

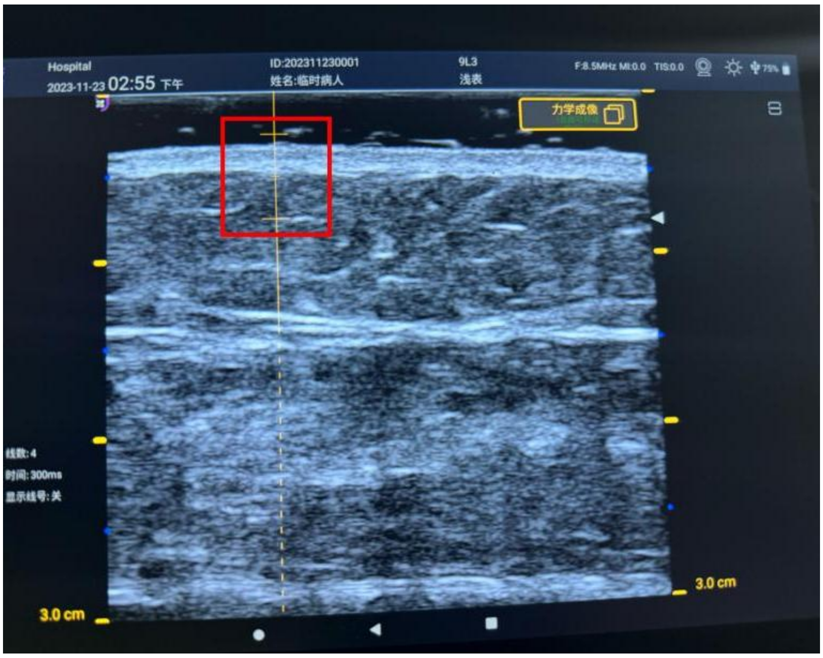

④After the settings are completed, click on the B button on the left sidebar of the main unit screen to enter B-mode ultrasound, and complete the preparation work.

Experimental Procedure

①Guide the subject to prepare for the test, evenly apply the pre-warmed coupling gel on the ultrasound transducer of the musculoskeletal mechanical quantitative detector, place the ultrasound transducer close to the measurement site so that the measurement point is directly below the front half of the transducer (the front is marked by a protrusion on one side of the ultrasound transducer), and observe the imaging effect in B-mode.

②Under clear B-ultrasound imaging conditions, use the ruler tool to measure and record the thickness from the skin surface to the corresponding muscle fascia layer to be tested.

③Turn on the excitation device switch, gently place the excitation end 3-6mm directly in front of the protrusion side of the transducer, ensuring that the excitation end is perpendicular to the imaging plane of the ultrasound transducer, as shown in the figure 3 below.

Figure 3

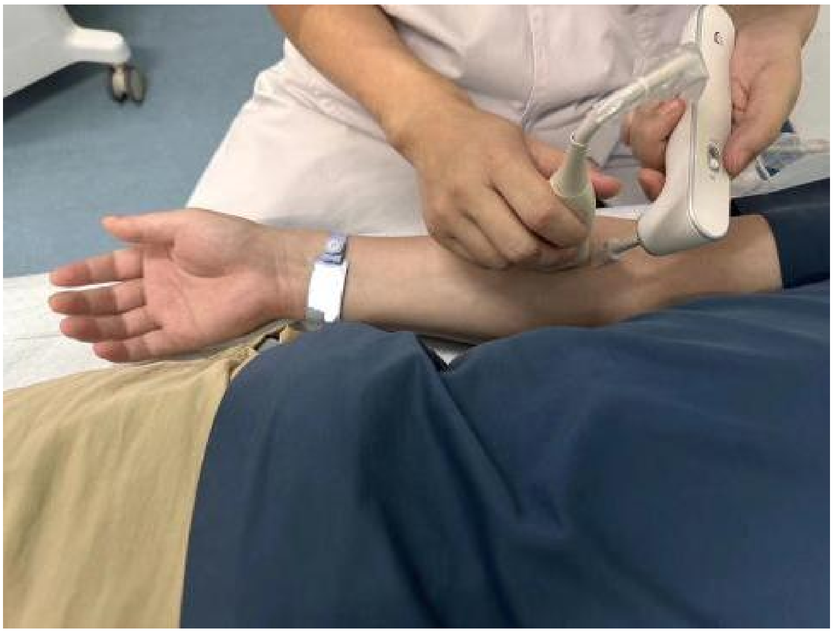

④Switch the musculoskeletal mechanical quantitative detector to E-mode to begin the measurement; the valid measurement values will be displayed on the screen interface.
⑤Record the measurement values in the corresponding sections of the experimental record form, completing this measurement experiment.
d: Standards for Data Collection:
①Locate the measurement point: Mark the measurement point on the muscle surface according to the study design to ensure consistency in measurement location each time.
②Apply coupling gel: Uniformly apply coupling gel to the measurement area to reduce signal attenuation.
③Each measurement should obtain no fewer than 5 valid measurement values, and the standard deviation of the measurement values should be less than 10% of the mean measurement value.

1.2.3 Grouping scheme

In a case-control design, the traditional rehabilitation assessments implemented for the knee joint include: 1. Knee joint muscle strength; 2. Range of motion (ROM) of the knee joint; 3. Lysholm score. The mechanical quantitative assessment team conducts mechanical quantitative evaluations on the knee joint.

1.2.4 Blind method

Due to the particularities of clinical controlled trials, a strict double-blind trial cannot be conducted. Therefore, based on the characteristics of this study, participants are aware of their group assignments, but evaluators are not informed of the specific research objectives and group allocation scheme. Data entry and statistical grouping personnel are also set up independently.

1.2.5 Statistical analysis scheme

The statistical analysis plan involves comparing the knee joint function assessment and mechanical quantitative modulus values, as well as the assessment results between the two sides. A statistical analysis software, SPSS 20.0, is used to establish

a database. Categorical data are represented as frequencies (percentages), and group comparisons are made using chi-square tests or exact probability methods. Continuous data are represented as means ± standard deviations, and group comparisons are made using t-tests, analysis of variance, or rank sum tests. Correlation analysis of related factors is performed using Spearman's correlation coefficient. A P-value< 0.05 is considered statistically significant.

1.2.6 Assessment tool description

Mechanical Quantitative Measurement Device (M5)

Manual Muscle Testing (MTT Muscle Strength Chart)

Range of Motion Assessment (using a Goniometer)

Knee Joint Function Assessment Scale (Lysholm Scale)
