# Supplementary material for: Application of mechanical quantitative techniques in postoperative rehabilitation assessment of anterior cruciate ligament reconstruction: A study protocol
Source: PLoS One. 2025 Aug 6;20(8):e0324663. doi: 10.1371/journal.pone.0324663 (PMC12327682; doi:10.1371/journal.pone.0324663)
Supplement: S4 Appendix — (DOCX) [file pone.0324663.s004.docx]

力学定量技术在前交叉韧带重建术后康复评估中的应用研究

研究方法

本课题以观察法为主，研究将围绕软组织力学定量检测技术展开，具体包括以下步骤：首先，收集前交叉韧带损伤患者的临床数据，包括病史、影像学检查等，确保样本的多样性和代表性。其次，利用软组织力学定量测量仪对患者膝关节进行定量、无损测量，获取骨骼肌力学状态的相关参数。在测量过程中，严格遵守操作规范，确保数据的准确性和可靠性。然后，对膝关节进行传统的康复评估。同时，结合生物力学的定量建模和表征方法，对测量数据进行深入分析，揭示前交叉韧带损伤患者膝关节功能康复程度和传统康复评估结果之间的定量对应关系。最后，根据分析结果，提出新的膝关节功能康复情况临床评价方法，并进行验证和评估。此外，还将收集患者的主观反馈，以进一步验证新评价方法的准确性和可行性。

研究实验方案

1.样本招募：

a.患者组纳入标准:

①明确单侧腿ACL损伤,关节镜下自体腘绳肌、髌腱ACL重建手术,无其他韧带合并损伤;

②患侧膝关节肿胀0级或1+级;

③术前损伤时间小于2个月;

④年龄 18—60 岁；

⑤签署康复治疗知情同意书,并积极配合治疗;

⑥治疗计划经湖南省康复医院伦理委员会批准；

b.排除标准:

①合并后交叉韧带断裂或半月板缝合;

②合并术后出现并发症等影响肢体锻炼者;

③既往有髋关节外伤的患者;

④伴高血压、糖尿病或其他器官慢性疾病者;

⑤合并心脏、脑部、肾及机体造血系统严重疾病者、精神疾病患者;

⑥合并任何不利于患者康复或者继续试验的情况。

d.样本量：

本研究中，膝关节功能lysholm评分表共8个变量， 基本功能评估包括肌力和关节活动范围2个变量，力学定量评估结果2个变量，本研究共 纳入 12 个研究变量，参考 Kendall 样本量估计法，纳入样本量为可变量数 5~10 倍，考虑10%的不合格率，总体样本量至少 66 例，综合实际情况，最终确定样本量为66例膝关节。

2.实施方法:

（1）数据统计与分析：①先由专人对符合纳入标准的病例进行评估并记录基线数据；②在康复治疗过程中、治疗后分别进行1次评估，并做好数据记录；③最后另设评估工作之外的专业人员进行数据统计和分析。

（2）关膝节功能评估：固定一位有经验的中级康复治疗师进行功能评估，评估内容包括：膝关节肌力、ROM、lysholm评分。

膝关节肌力评分：检查时，患者处于不同的受检位置，对受试的肌肉或肌群在减重、抗重力或抗阻力的状况下做一定动作，并使动作达到最大的活动范围。根据肌肉完成动作的情况，按肌力分级标准来评定肌力的级别，主要分为6个级别，即0级、1级、2级、3级、4级、5级。其中5级是正常水平。

lysholm评分：①‌疼痛评分‌：0-25分。②不稳定评分‌：0-25分。③下蹲评分‌：0-5分。④绞索评分‌：0-15分。⑤上楼评分：0-10分。⑥支持评分：0-5分。⑦肿胀评分：0-10分。⑧跛行评分：0-5分。

（3）软组织力学定量评估：固定一位具有５年以上工作经验的医师进行软组织力学定量评估，评估内容包括软组织力学定量检测：

①股直肌剪切模量G（modulus of rigidity，G）；②腘绳肌肌剪切模量G。

具体操作方法：

a：测量方法：利用软组织力学定量检测仪（M5），测量受试者股直肌和腘绳肌的力学定量表征（剪切模量G）。

b：受试者体位：患者采取仰卧位，双下肢放松。

c：操作SOP：

（1）规程：实验器材、耗材与纸质资料

①实验器材：软组织力学定量检测仪，包含主机、9L3-8.5MHZ-线阵探头、机械激励模组。

②耗材：超声耦合剂。

③纸质资料：受试者知情同意书、受试者信息采集表、实验记录表。

④其它实验用具和耗材（包括常规医疗床、卷尺、插线板、超声专用纸、记号笔、发热贴、收纳箱、矮凳等）。

（2）实验前准备

①耦合剂预热：实验正式开始前1小时，将一片发热贴贴在耦合剂瓶身上，使正式实验是耦合剂温度与人体体温相当；

②纸质资料准备：根据受试者数量打印纸质资料，每人一份；

③受试者知情告知：由实验者对受试者进行知情告知（包括实验过程、获益与风险、保密方案等），受试者充分知情同意后签署知情同意书；

④受试者填写信息采集表；

（3）仪器准备

①将软组织力学定量检测仪相关配件摆放在实验台上，确认主机与探头连接正常；插排接电源置于实验箱中，主机连接插排。长按软组织力学定量检测仪主机侧方开机键，直至电源指示灯亮起，打开app，并确认登录。

②点选主机屏幕左侧边栏E按钮，进入力声采集模式，查看参数如下表所示：

| 参数 | 值 |
| --- | --- |
| 频率 | 6.0M |
| 线数 | 4 |
| 范围 | 5mm |
| 时间 | 300ms |
| 位置预制 | 2 |
| 显示线号 | 关 |

③调整测量点位卡尺的深度位置，将采集深度范围起点调整至探头下方真皮层处如图所示：


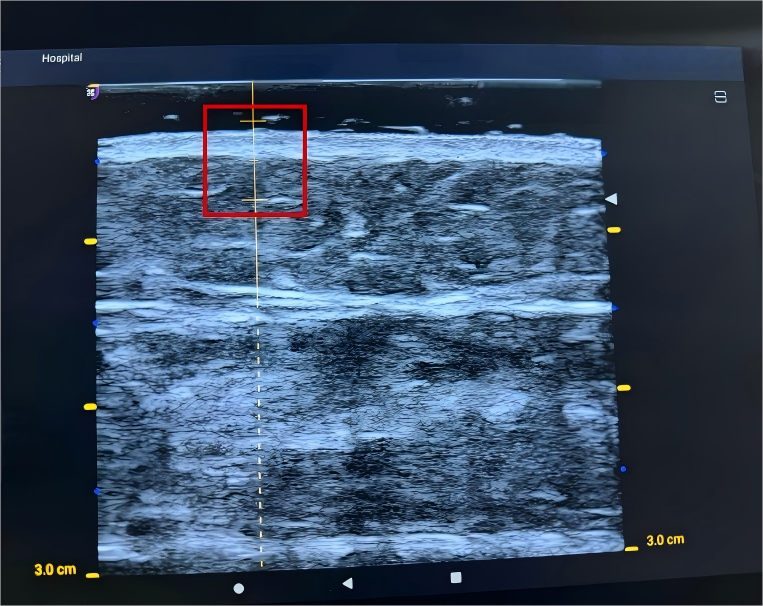


④设置完成后点选主机屏幕左边栏B按钮，进入B超模式，完成准备工作。

（4）实验流程

①指导受试者做好测试准备，将预热的耦合剂均匀地涂在软组织力学定量检测仪的超声探头上，将超声探头贴近测量部位，使测量点位于前半个探头的正下方（超声探头一侧有突起标记为前），在B模式下观察成像效果。

②在B超图像清晰的条件下，点击尺子工具测量皮肤表面到相应待测肌肉筋膜层的厚度并记录。

③打开激励装置开关，将激励端轻放在探头突起侧正前方3-6mm处，激励端直线与超声探头成像平面保持垂直，如下图。


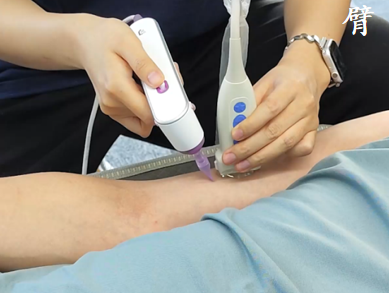


④将软组织力学定量检测仪切换至E模式，开始测量，有效测量的数值将在屏幕界面中显示。

⑤在实验记录表中相应位置记录本次测量值，完成本次测量实验。

d：采集数据的标准：

①定位测量点：根据研究设计，在肌肉表面标记测量点，确保每次测量位置一致。

②涂抹耦合剂：在测量区域均匀涂抹耦合剂，减少信号衰减。

③每次测量要得到不少于5个有效测量值，测量值的标准差要小于10%的测量均值。

3.分组方案:

按照病例-对照设计，其中传统评估组对膝关节评估的实施的评估包括：1.膝关节肌力；2.膝关节活动范围；3.lysholm评分。力学定量组队膝关节实施力学定量评估。

4.盲法:

由于临床对照试验的特殊性，无法进行严格意义的双盲试验，故根据本研究的特征设置为参与者知道分组结果，但评估者不知道具体研究目的和分组方案。数据录入和统计分组人员也独立设置。

5.统计分析方案

对患者膝关节功能评估与力学定量模量值之间以及双侧的评估结果进行对比，采用统计分析软件SPSS20.0建立数据库，计数资料采用频数（百分率）表示，组间比较采用χ2检验或确切概率法；计量资 料采用均数±标准差表示，组间比较采用t检验,方差分析或秩和检验,相关因素分析采用Spearman相关系数。P<0.05认为差异有统计学意义 。

6.评估工具具体描述：

力学定量测量仪（M5）

徒手肌力评估（MTT肌力量表）

关节活动范围评估（使用量角器）

膝关节关节功能评估量表（lysholm）

⑦预期结果：

力学定量技术可提供客观的量化数据，亦可揭示其结果与传统功能评估方法的相关性，可作为评估膝关节功能的有效工具。

Application of Mechanical Quantitative Techniques in Postoperative Rehabilitation Assessment of Anterior Cruciate Ligament Reconstruction：a study protocol

**Methods**

This study primarily employs an observational approach, focusing on musculoskeletal mechanical quantitative detection technology, and includes the following steps: Firstly, collect clinical data from patients with anterior cruciate ligament injuries, including medical history, imaging examinations, etc., to ensure the diversity and representativeness of the sample. Secondly, use musculoskeletal mechanical quantitative measurement devices to conduct quantitative, non-destructive measurements of the patient's knee joint to obtain relevant parameters of the mechanical state of skeletal muscles. During the measurement process, strictly adhere to operational protocols to ensure the accuracy and reliability of the data. Then, conduct traditional rehabilitation assessments of the knee joint. Concurrently, combine quantitative modeling and characterization methods of biomechanics to deeply analyze the measurement data, revealing the quantitative correlation between the degree of knee joint functional recovery in patients with anterior cruciate ligament injuries and the results of traditional rehabilitation assessments. Finally, based on the analysis results, propose a new clinical evaluation method for knee joint functional recovery and conduct validation and assessment. Additionally, collect subjective feedback from patients to further verify the accuracy and feasibility of the new evaluation method.

**Experimental study design**

**1.Sample recruitment**

Inclusion criteria:

①Unilateral leg ACL injury, arthroscopic autologous hamstring or patellar tendon ACL reconstruction surgery, with no other ligamentous injuries involved;

②Swelling of the affected knee joint is grade 0 or 1+;

③Time from injury to surgery is less than 2 months;

④Ages between 18 and 60 years;

⑤Signed an informed consent form for rehabilitation treatment and actively cooperate with the treatment;

⑥The treatment plan is approved by the Ethics Committee of the Hunan Provincial Rehabilitation Hospital;

Exclusion criteria

①Concurrent posterior cruciate ligament rupture or meniscal repair;

②Concurrent postoperative complications affecting limb exercise;

③Patients with a history of hip joint trauma;

④Individuals with hypertension, diabetes, or other chronic diseases of organs;

⑤Individuals with concurrent severe diseases of the heart, brain, kidneys, and hematopoietic system, and patients with mental illnesses;

⑥Concurrent with any conditions that are detrimental to patient recovery or continuation of the trial.

Sample size

In this study, the Lysholm knee score questionnaire comprises a total of 8 variables, basic functional assessment includes 2 variables: muscle strength and range of motion, mechanical quantitative assessment results include 2 variables, a total of 12 research variables are included in this study, referring to Kendall's sample size estimation method, the sample size included is 5 to 10 times the number of variables, considering a 10% rate of ineligible cases, the total sample size should be at least 66 cases, according to the actual situation, the final sample size is determined to be 66 knee joints.

**2.Implementation method**

(1)Data Statistics and Analysis:①Firstly, a designated person evaluates the cases that meet the inclusion criteria and records the baseline data;②One assessment is conducted during the rehabilitation process and after treatment, with proper data recording;③Finally, a professional separate from the assessment work performs data statistics and analysis.(2) Knee Joint Function Assessment: A fixed experienced intermediate rehabilitation therapist conducts the functional assessment, which includes: knee joint muscle strength, ROM, and Lysholm score.

(2) Knee Joint Muscle Strength Grading: During the examination, the patient is placed in different positions to be tested, and the targeted muscles or muscle groups perform specific movements under conditions of weight reduction, gravity resistance, or resistance, achieving the maximum range of motion. Based on the muscle's ability to perform the movement, muscle strength is graded according to the grading standards, which are mainly divided into 6 levels: 0, 1, 2, 3, 4, and 5. Level 5 represents normal strength.

Lysholm Score:①Pain Score: 0-25 points.②Instability Score: 0-25 points.③Squatting Score: 0-5 points.④Locking Score: 0-15 points.⑤Climbing Stairs Score: 0-10 points.⑥Support Score: 0-5 points.⑦Swelling Score: 0-10 points.⑧Gait Score: 0-5 points.

(3) Musculoskeletal Mechanical Quantitative Assessment: A physician with over 5 years of experience is designated to conduct musculoskeletal mechanical quantitative assessments, The assessment includes musculoskeletal mechanical quantitative testing:①Shear modulus of the rectus femoris muscle (modulus of rigidity, G);②Shear modulus of the hamstring muscles (modulus of rigidity, G).

Specific operation method:

a: Measurement Method: Utilize a musculoskeletal mechanical quantitative detector (M5) to measure the mechanical quantitative characteristics (shear modulus G) of the subject's rectus femoris and hamstring muscles.

b: Subject Position: The patient is in a supine position with both lower limbs relaxed.

c: Operating SOP(Standard Operating Procedure):

Protocol: Experimental Equipment, Consumables, and Paper Documents:

①Experimental Equipment: Musculoskeletal mechanical quantitative detector, including the main unit, 9L3-8.5MHZ linear array transducer, and mechanical excitation module.

②Consumables: Ultrasound coupling gel.

③Paper Documents: Informed consent form for subjects, subject information collection form, and experimental record form.

④Other Experimental Tools and Consumables (including a standard medical bed, measuring tape, power strip, ultrasound-specific paper, marker, heat patch, storage box, low stool, etc.).

Pre-experimental Preparation:

①Coupling Gel Preheating: One hour before the official start of the experiment, attach a heat patch to the coupling gel bottle to warm it to a temperature similar to body temperature for the formal experiment;

②Paper Document Preparation: Print paper documents according to the number of subjects, one per person;

③Subject Informed Consent: The experimenter informs the subjects about the experimental process, benefits, risks, confidentiality plan, etc., and after being fully informed, the subjects sign the informed consent form;

④Subject Completes Information Collection Form;

Instrument Preparation

①Place the musculoskeletal mechanical quantitative detector and its accessories on the experimental table, ensuring the main unit is properly connected to the transducer; place the power strip in the experiment box, connect the main unit to the power strip. Press and hold the power button on the side of the musculoskeletal mechanical quantitative detector until the power indicator light turns on, open the app, and confirm login.

②Tap the E button on the left sidebar of the main unit screen to enter the force-sound acquisition mode, and check the parameters as shown in the table 1 below:

| **Table 1. Parameters** | |
| --- | --- |
| Parameter | Value |
| Frequency | 6.0M |
| Line Count | 4 |
| Range | 5mm |
| Time | 300ms |
| Position Preset | 2 |
| Display Line Number | Off |

③Adjust the depth position of the measurement point caliper to set the starting point of the acquisition depth range just below the dermis layer under the transducer as shown in the figure 2:

| **Figure 2** |
| --- |
| 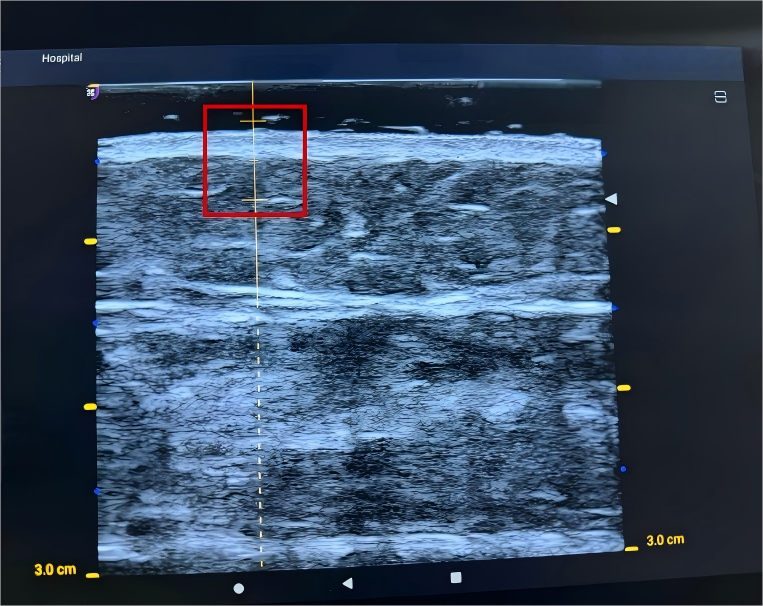 |

④After the settings are completed, click on the B button on the left sidebar of the main unit screen to enter B-mode ultrasound, and complete the preparation work.

Experimental Procedure

①Guide the subject to prepare for the test, evenly apply the pre-warmed coupling gel on the ultrasound transducer of the musculoskeletal mechanical quantitative detector, place the ultrasound transducer close to the measurement site so that the measurement point is directly below the front half of the transducer (the front is marked by a protrusion on one side of the ultrasound transducer), and observe the imaging effect in B-mode.

②Under clear B-ultrasound imaging conditions, use the ruler tool to measure and record the thickness from the skin surface to the corresponding muscle fascia layer to be tested.

③Turn on the excitation device switch, gently place the excitation end 3-6mm directly in front of the protrusion side of the transducer, ensuring that the excitation end is perpendicular to the imaging plane of the ultrasound transducer, as shown in the figure 3 below.

| **Figure 3** |
| --- |
| 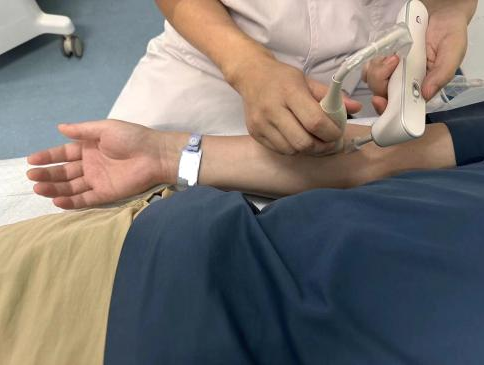 |

④Switch the musculoskeletal mechanical quantitative detector to E-mode to begin the measurement; the valid measurement values will be displayed on the screen interface.

⑤Record the measurement values in the corresponding sections of the experimental record form, completing this measurement experiment.

d: Standards for Data Collection:

①Locate the measurement point: Mark the measurement point on the muscle surface according to the study design to ensure consistency in measurement location each time.

②Apply coupling gel: Uniformly apply coupling gel to the measurement area to reduce signal attenuation.

③Each measurement should obtain no fewer than 5 valid measurement values, and the standard deviation of the measurement values should be less than 10% of the mean measurement value.

**3.Grouping scheme**

In a case-control design, the traditional rehabilitation assessments implemented for the knee joint include: 1. Knee joint muscle strength; 2. Range of motion (ROM) of the knee joint; 3. Lysholm score. The mechanical quantitative assessment team conducts mechanical quantitative evaluations on the knee joint.

**4.Blind method**

Due to the particularities of clinical controlled trials, a strict double-blind trial cannot be conducted. Therefore, based on the characteristics of this study, participants are aware of their group assignments, but evaluators are not informed of the specific research objectives and group allocation scheme. Data entry and statistical grouping personnel are also set up independently.

**5.Statistical analysis scheme**

The statistical analysis plan involves comparing the knee joint function assessment and mechanical quantitative modulus values, as well as the assessment results between the two sides. A statistical analysis software, SPSS 20.0, is used to establish a database. Categorical data are represented as frequencies (percentages), and group comparisons are made using chi-square tests or exact probability methods. Continuous data are represented as means ± standard deviations, and group comparisons are made using t-tests, analysis of variance, or rank sum tests. Correlation analysis of related factors is performed using Spearman's correlation coefficient. A P-value< 0.05 is considered statistically significant.

**6.Assessment tool description**

Mechanical Quantitative Measurement Device (M5)

Manual Muscle Testing (MTT Muscle Strength Chart)

Range of Motion Assessment (using a Goniometer)

Knee Joint Function Assessment Scale (Lysholm Scale)
